# Supplementary material for: Single Cell Transcriptome Analysis of Niemann–Pick Disease, Type C1 Cerebella
Source: Int J Mol Sci. 2020 Jul 28;21(15):5368. doi: 10.3390/ijms21155368 (PMC7432835; doi:10.3390/ijms21155368)
Supplement: Supplementary file 1 [file ijms-21-05368-s001.zip › Supplemental 7-4.pdf]

Supplemental Material

Single cell transcriptome analysis of Niemann-Pick disease, type C1 cerebella

Cougnoux et al.

Supplemental figures and tables

		Page
Figure S1	t-SNE plots 7-week data	1
Figure S2	Microglia	2
Figure S3	Vascular cells	3
Figure S4	Glial cells	4
Figure S5	Purkinje neurons	6
Figure S6	Protein validation	7
Figure S7	T-SNE plots 3-week data	8
Table S1	Differentially expressed genes	9
Table S2	Pathway Analysis	See Excel file
Table S3	Antibodies, primers and reagents	18

Figure S1

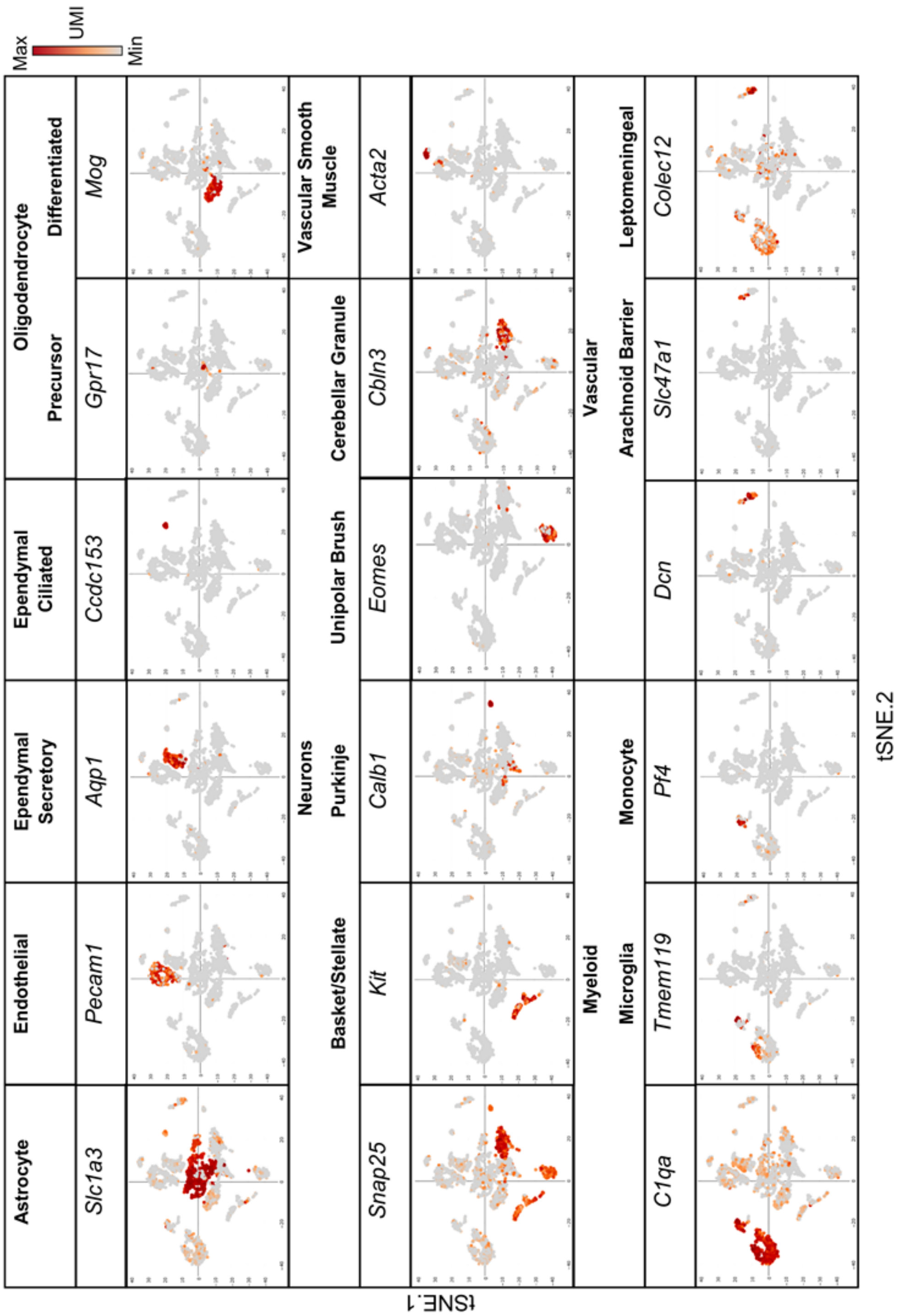

Figure S1. t-SNE plots of the different cell types identified in cerebellar tissue from 7-week-old *Npc1*$^{+/+}$ and *Npc1*$^{-/-}$ mice. UMI: Unique Molecular Identifier; Max: maximum; Min: minimum.

Figure S2

A.

B.

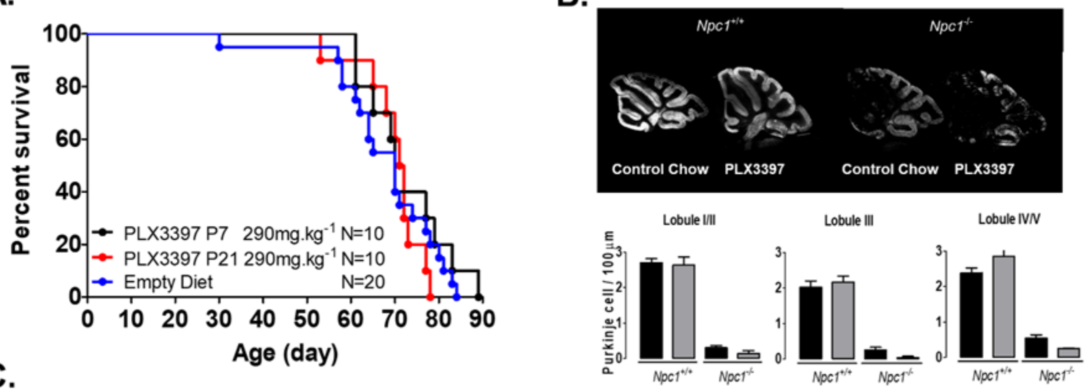

C.

KEGG	Description	p-value	pAdj-value
mmu03010	Ribosome	1.22E-11	5.38E-10
mmu05016	Huntington disease	9.67E-12	5.38E-10
mmu05012	Parkinson disease	6.42E-12	5.38E-10
mmu05010	Alzheimer disease	4.08E-12	5.38E-10
mmu04932	Non-alcoholic fatty liver disease	1.03E-11	5.38E-10
mmu04145	Phagosome	0.000457	0.012062
mmu04142	Lysosome	1.89E-05	0.000623
mmu00010	Glycolysis / Gluconeogenesis	0.00079	0.018958

D.

E.

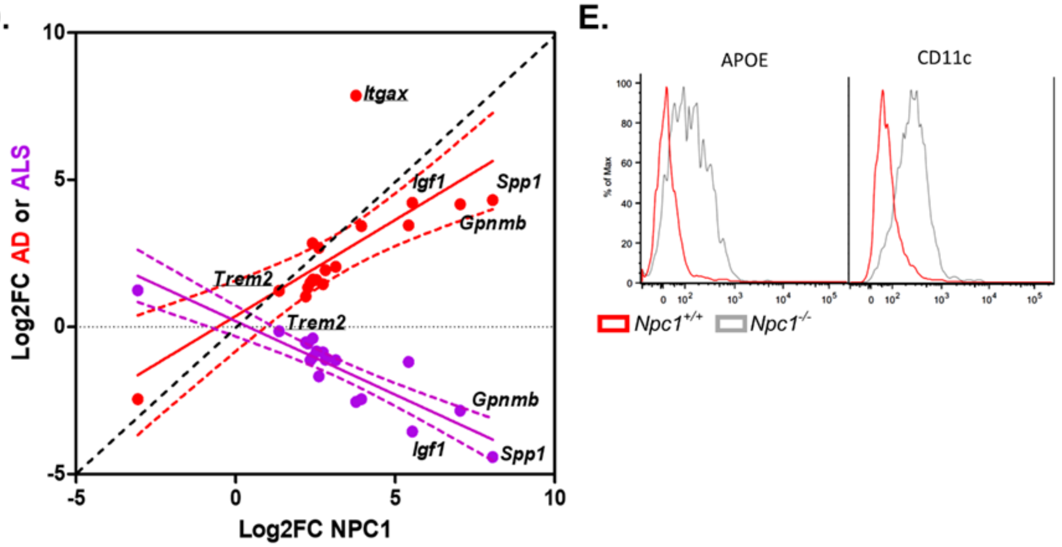

Figure S2. (A) Kaplan-Meier survival graphic of 290mg.kg^{-1} PLX3397 treatment starting at day of life 7 (Black) 21 (Red) and control diet (Blue). **(B)** Purkinje neuron pathology analysis at 7 weeks of age of the mice starting at day of life 21. **(C)** KEGG pathway analysis demonstrated similarity between altered gene expression in Npc1-/- microglia and microglia from mouse models of common neurodegenerative diseases. **(D)** Correlation of **D**isease **A**ssociated **M**icroglia (DAM) profile between *Npc1*$^{-/-}$ and either Alzheimer disease (Red) or Amyotrophic Lateral Sclerosis (Purple) mice. Data for Alzheimer and Amyotrophic Lateral Sclerosis are from Keren-Shaul et al. [36]. **(D)** Flow cytometry validation of increased expression of DAM microglia markers APOE and CD11C overexpression in *Npc1*$^{-/-}$ (gray) compared to *Npc1*$^{+/+}$ (red) 7-week-old mice cerebellar microglia.

Figure S3

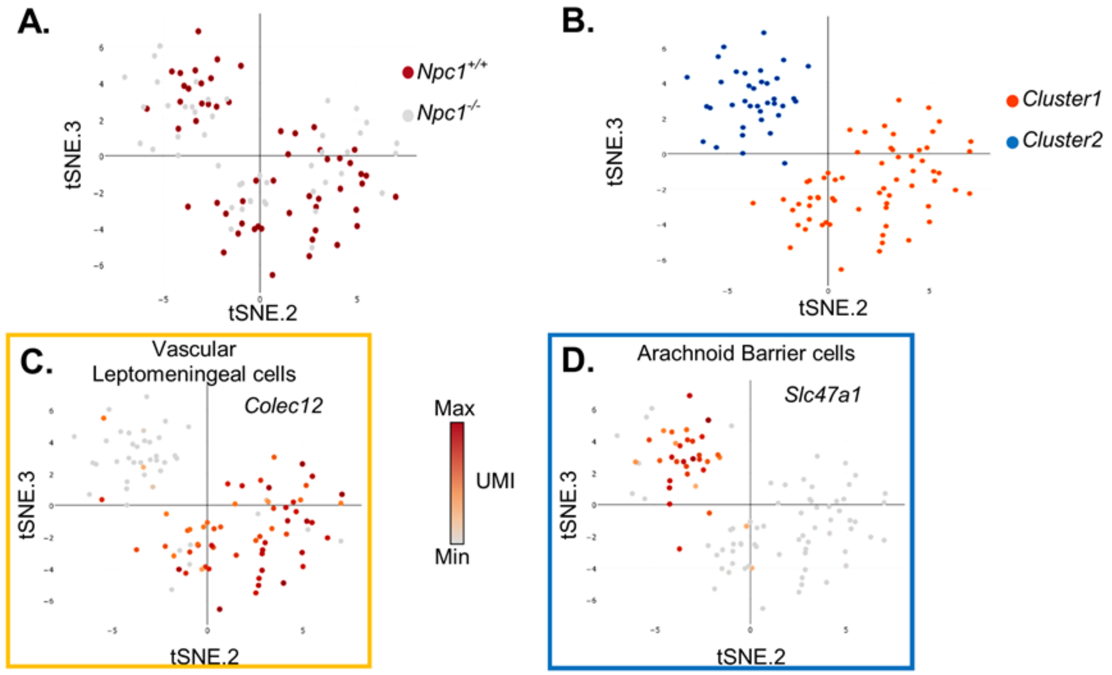

Figure S3. (A) t-SNE plot of cerebellar vascular cells that were not identified as vascular smooth muscle or endothelial cells from *Npc1*$^{+/+}$ (red) and *Npc1*$^{-/-}$ (gray) 7-week-old mice. **(B)** These cells formed two clusters which could be identified as arachnoid barrier cells (cluster 1, orange) and vascular leptomeningeal cells (cluster 2, blue) based upon expression of *Colec12* **(C)** in vascular leptomeningeal cell and *Slc47a1* **(D)** in arachnoid barrier cells.

Figure S4

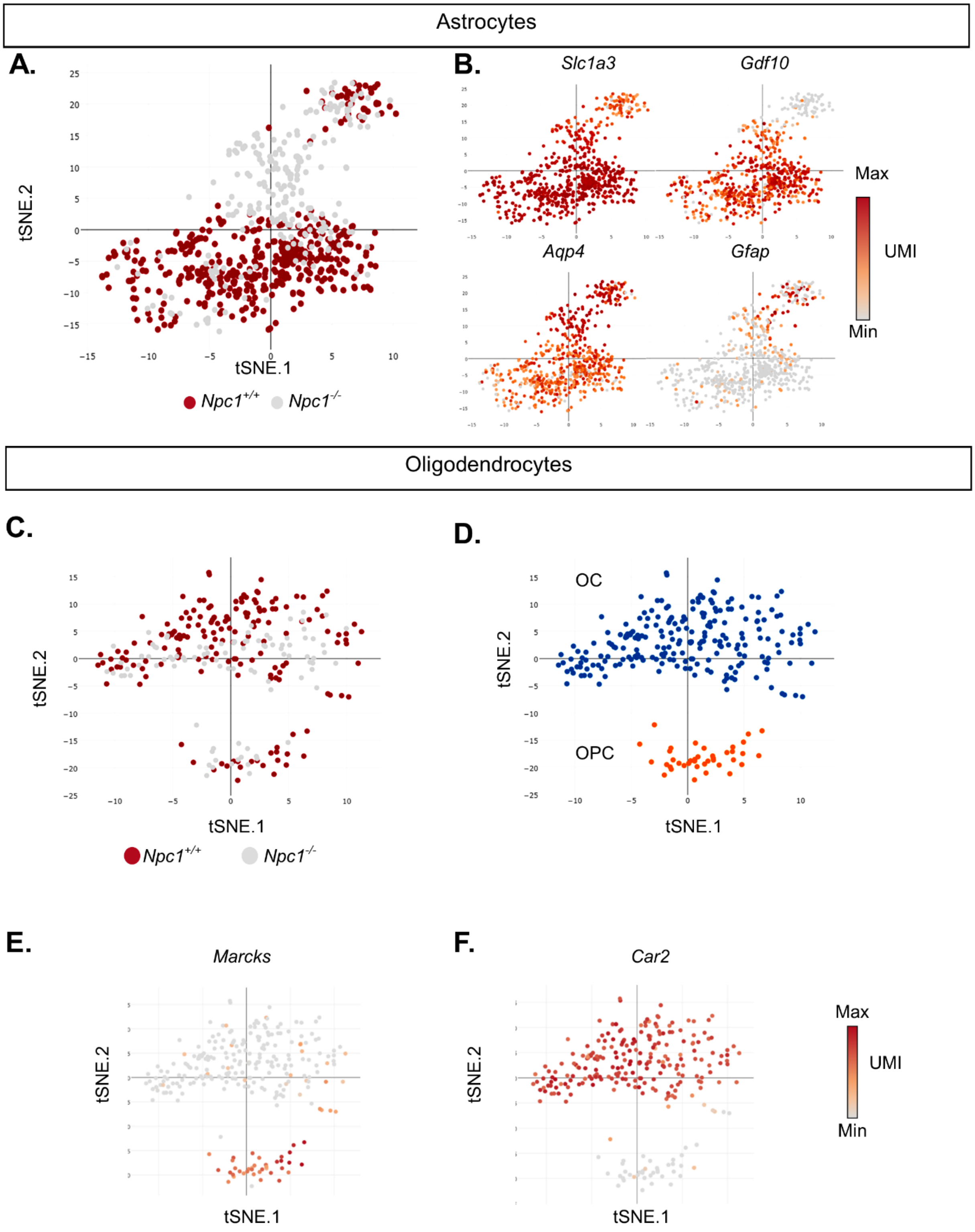

Astrocytes
A.
tSNE.2
tSNE.1
Npc1+/+
Npc1-/-
B.
Slc1a3
Gdf10
Aqp4
Gfap
Max
UMI
Min
Oligodendrocytes
C.
tSNE.2
tSNE.1
Npc1+/+
Npc1-/-
D.
OC
OPC
tSNE.2
tSNE.1
E.
Marcks
tSNE.2
tSNE.1
F.
Car2
tSNE.2
tSNE.1
Max
UMI
Min

Figure S4 (continued)

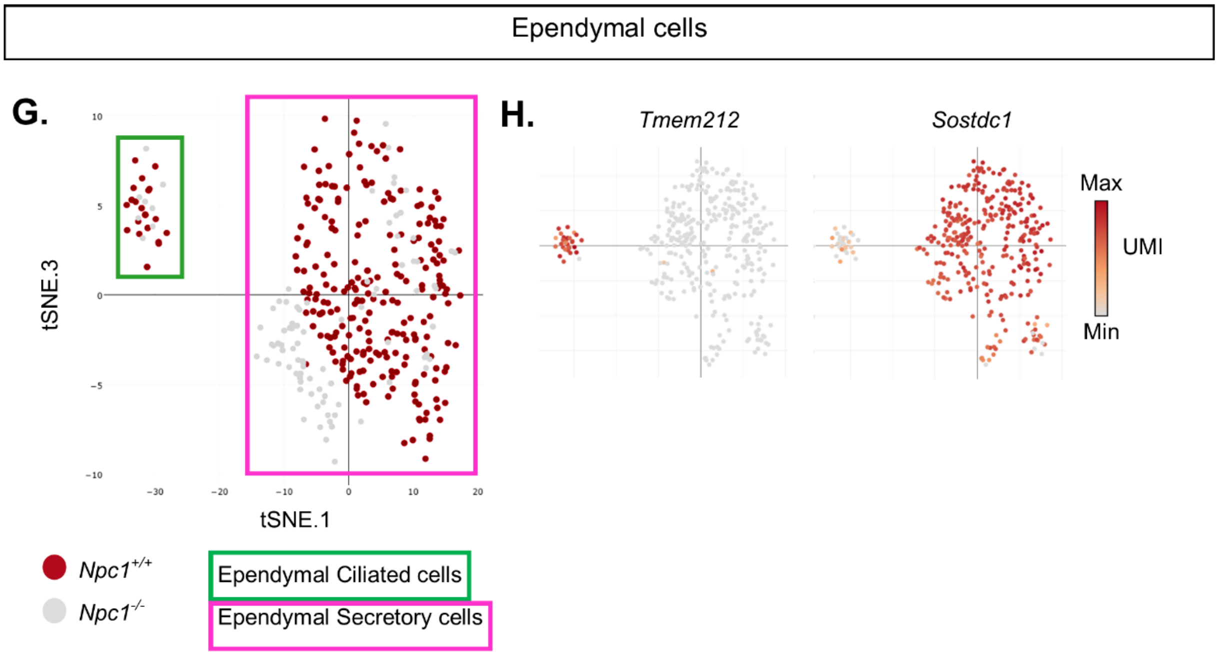

Figure S4. (A) t-SNE plot of astrocytes from *Npc1*$^{+/+}$ (red) and *Npc1*$^{-/-}$ (gray) 7-week-old mice. **(B)** t-SNE plots of *Slc1a3*, *Gfap*, *Aqp4* and *Gdf10* expression levels in astrocytes. **(C)** t-SNE plot of the oligodendrocytes from *Npc1*$^{+/+}$ (red) and *Npc1*$^{-/-}$ (gray) 7-week-old mice. **(D)** K-Mean clustering on t-SNE plot of the oligodendrocytes from both genotypes. OC: oligodendrocytes (blue) and OPC: oligodendrocyte precursors (orange). t-SNE plots of *Marcks* **(E)** and *Car2* **(F)** expression in oligodendrocyte precursors and oligodendrocytes, respectively. **(G)** t-SNE plot of ependymal cells from *Npc1*$^{+/+}$ (red) and *Npc1*$^{-/-}$ (gray) 7-week-old mice. Ependymal ciliated cells (green box) and ependymal secretory cells (pink box). (H) t-SNE plots of *Tmem212* and *Sostdc1* expression by ependymal cells. UMI: Unique Molecular Identifier; Max: maximum; Min: minimum.

Figure S5

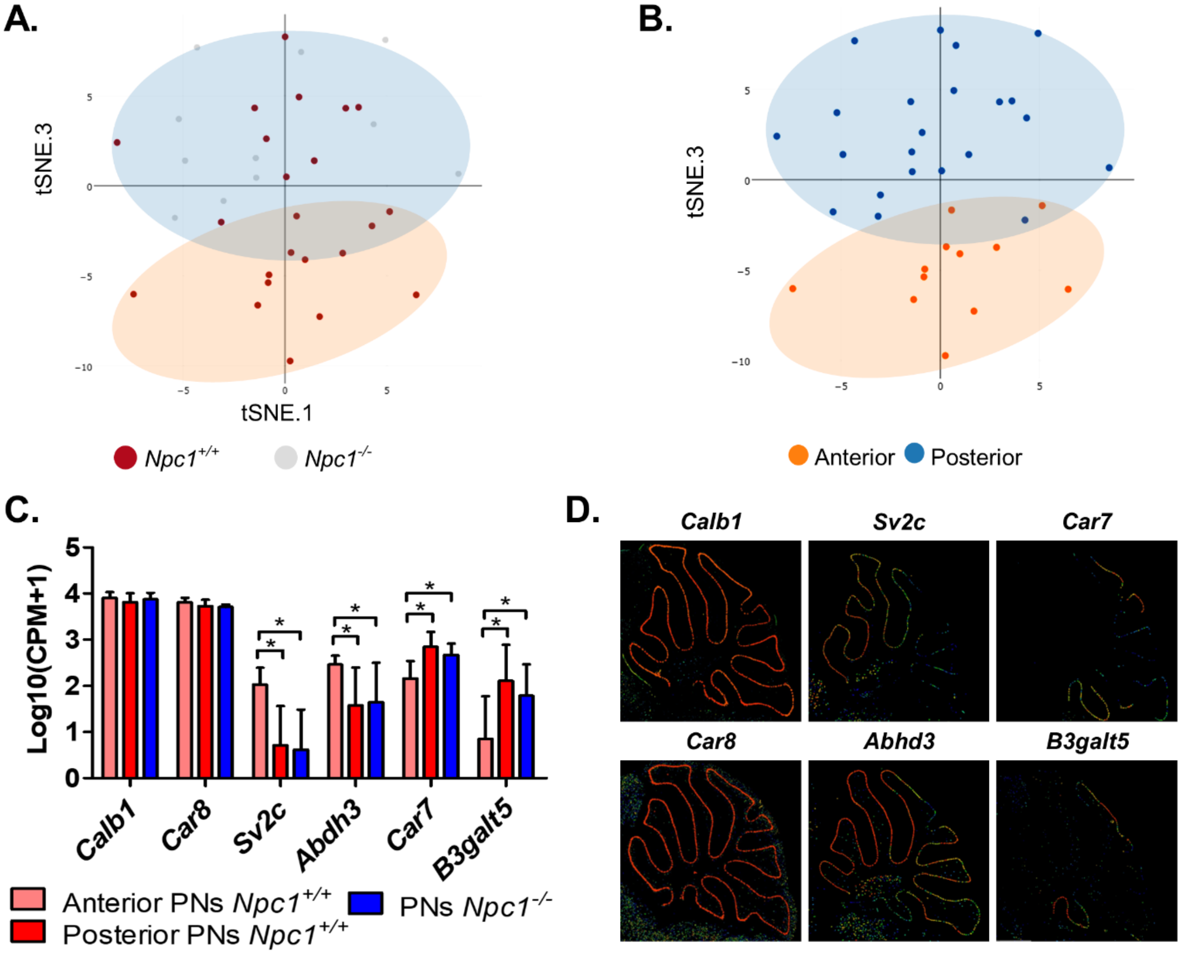

Figure S5 extended Figure 5: **(A)** t-SNE plot of the Purkinje cells from *Npc1*$^{+/+}$ (red) and *Npc1*$^{-/-}$ (gray) 7-week-old mice. The light blue ellipse surrounds the posterior Purkinje cells. The light orange ellipse surrounds the anterior Purkinje cells. **(B)** K-Mean clustering on t-SNE plot of the Purkinje cells from both genotypes of 7-week-old mice. The 2 clusters separate cells from the posterior (blue) and anterior (orange) cerebellum. The light blue ellipse surrounds the posterior Purkinje cells. The light orange ellipse surrounds the anterior Purkinje cells. **(C)** Expression level of transcripts used to differentiate anterior and posterior Purkinje neurons. **(D)** Corresponding *in situ* expression of genes used to identify anterior and posterior Purkinje neurons from the Allen brain atlas (https://portal.brain-map.org/).

Figure S6

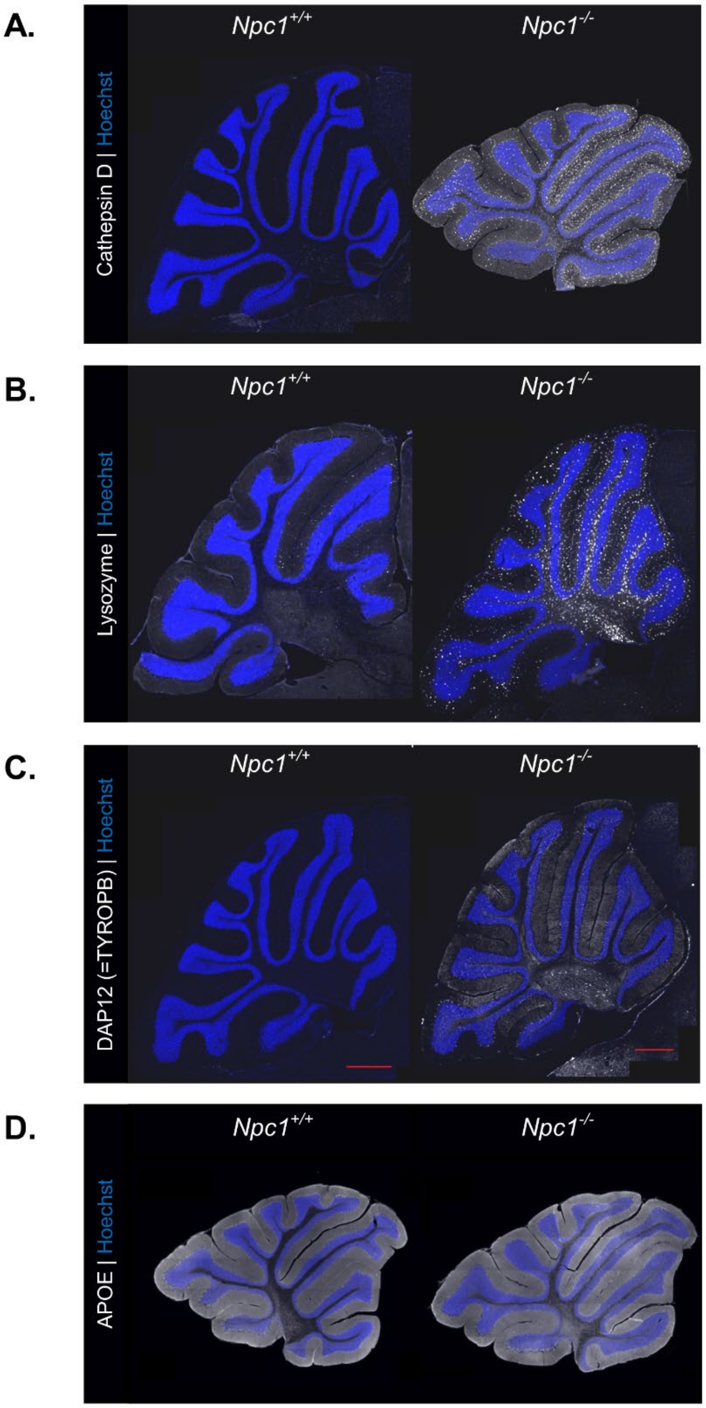
A.
Npc1+/+
Npc1-/-
Cathepsin D | Hoechst
B.
Npc1+/+
Npc1-/-
Lysozyme | Hoechst
C.
Npc1+/+
Npc1-/-
DAP12 (=TYROPB) | Hoechst
D.
Npc1+/+
Npc1-/-
APOE | Hoechst

Figure S6. Representative immunostaining of 7-week-old *Npc1*$^{+/+}$ and *Npc1*$^{-/-}$ cerebellum stained for Cathepsin D (A), Lysozyme (B), DAP12 (C) and APOE (D). Nuclei were counterstained with Hoechst (Blue).

Figure S7

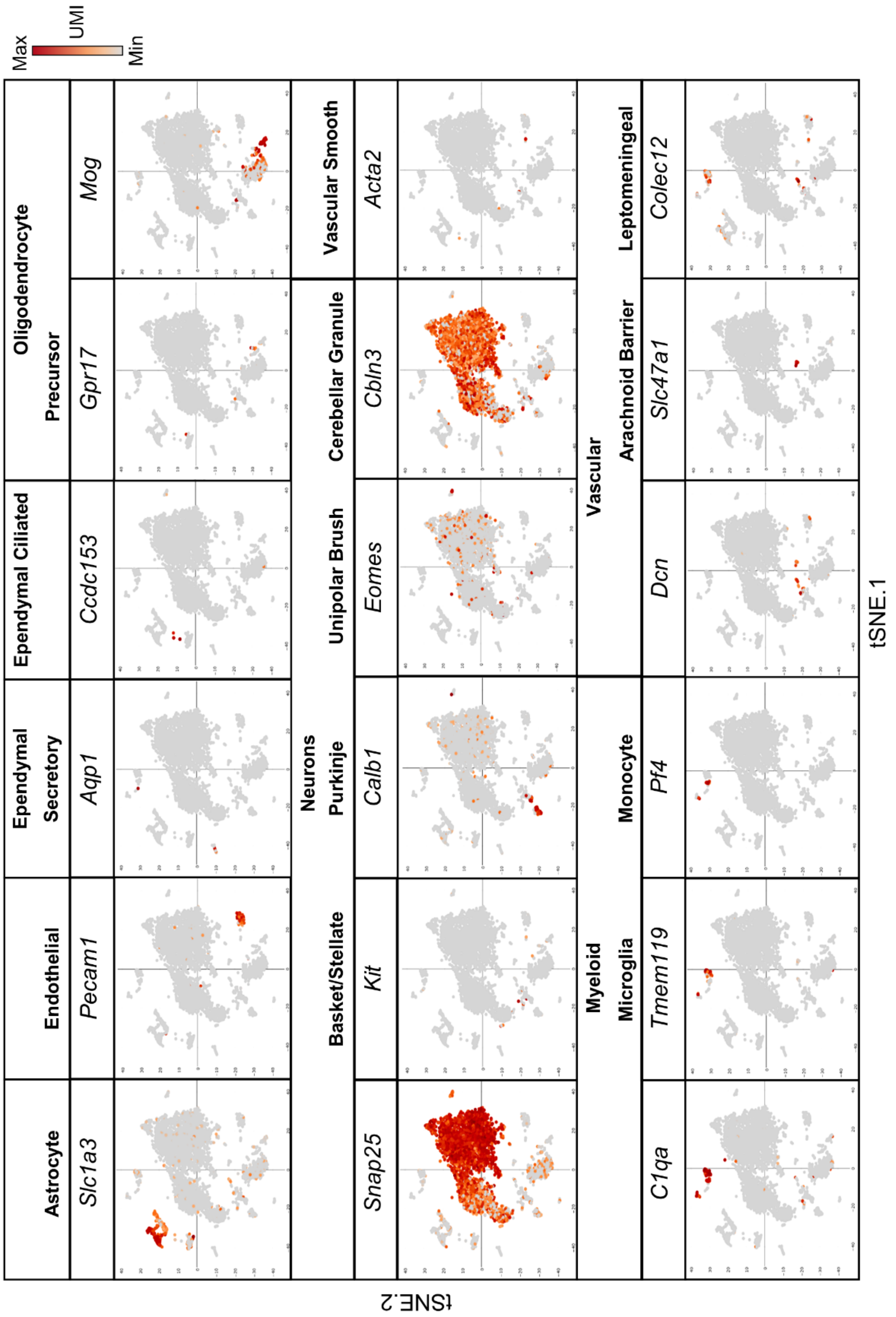

Figure S7. t-SNE plots of the different cell types identified in cerebellar tissue from 3-week-old *Npc1*$^{+/+}$ and *Npc1*$^{-/-}$ mice. UMI: Unique Molecular Identifier; Max: maximum; Min: minimum.

Table S1

	Glial Cells					Vascular Cells			Neurons					
**Gene**	Astrocytes **(A)**	Ependymal ciliated cells **(ECC)**	Ependymal Secretory cells **(ESC)**	Oligodendrocytes **(O)**	Oligodendrocytes Precursor **(OP)**	Endothelial cells **(EC)**	Vascular Leptomeningeal cells **(VLC)**	Vascular Smooth muscle cells **(VSM)**	Basket/Stellate cells **(B/S)**	Cerebellar granule cells **(GC)**	Interneurons **(IN)**	Purkinje neurons **(PN)**	Unipolar Brush Cell **(UBC)**	**Gene description and relevance to NPC1**

7-week-old NPC1 mutant expression

Agt	↑													*Agt*, encodes angiotensinogen precursor previously described to be a mature astrocyte marker important for blood-brain-barrier integrity [1].
Apoe			↑	↑	↑		↑	↑		↑	↑	↑	↑	*Apoe* encodes Apolipoprotein E, the main extracellular cholesterol carrier in the central nervous system. Variants of apolipoprotein E have been associated with Alzheimer and Niemann-Pick type C disease severity [2, 3].
Aqp4	↑													*Aqp4* encodes Aquaporin 4, an intrinsic plasma membrane protein that functions as water-selective channels and controls brain water homeostasis and blood-brain permeability [4].
Arhgap31					↑									*Arhgap31* encodes Rho GTPase-activating protein 31 which interacts with Rho GTPases RAC1 and CDC42 [5]. Endothelial cell expression of *Arhgap31* is important for vascular development and VEGF mediated angiogenesis, but its function in oligodendrocytes is not known [5]. Pathogenic variants of *ARHGAP31* are causative of Adams-Oliver syndrome [6].

Table S1 (continued)

Gene	A	ECC	ESC	O	OP	EC	VLC	VSM	B/S	GC	IN	PN	UBC	Gene description and relevance to NPC1
B2m			↑	↑								↑		*B2m* encodes beta 2 microglobulin, a protein essential for the expression of properly folded MHC-I protein [7].
C1qa		↑	↑									↑		***C1qa, C1qb* and *C1qc* encode subunits of complement C1.** A previous study by Lopez *et al*., demonstrated the lack of a role played by *C1qa* in *Npc1*$^{-/-}$ cerebellar disease progression [8].
C1qb			↑											
C1qc			↑											
Cd63				↑										*Cd63*/CD63 (LAMP3) is a member of the transmembrane 4 superfamily or tetraspanins expressed in late endosomes, lysosomes and at the surface of exosomes [9]. Loss of *Npc1* has been studied in oligodendrocytes and shown to enhance exosome secretion. Thus, increased expression of Cd63 may be a cellular response to decrease unesterified cholesterol storage. Strauss *et al*., [10] previously demonstrated in NPC1 that exosomal release of unesterified cholesterol can bypass the intracellular trafficking defect to maintain cellular cholesterol homeostasis.
Ctla2a						↑								*Ctla2a* encodes Cytotoxic T-Lymphocyte Antigen-2α, a protein with immunomodulatory property in addition to its Cathepsin L like cysteine protease inhibition function [11].
Ctsb	↑			↑		↑								*Ctsb* encodes the lysosomal cysteine protease cathepsin B. Cathepsin B appears to be neuroprotective in NPC1 given that loss of cathepsin B function in *Npc1*$^{-/-}$:*Ctsb*$^{-/-}$ mice leads to a more severe NPC1 phenotype [12].
Ctsd	↑			↑	↑	↑		↑	↑	↑	↑	↑	↑	*Ctsd* encodes Cathepsin D, a lysosomal aspartyl protease mutated in type 10 neuronal ceroid lipofuscinosis [13]. It has previously been reported to be overexpressed in NPC1 cerebellum over the course of the disease in microglia and neurons [14].

Table S1 (continued)

Gene	A	ECC	ESC	O	OP	EC	VLC	VSM	B/S	GC	IN	PN	UBC	Gene description and relevance to NPC1
Ctsl			↑											*Ctsl* encodes the protein Cathepsin L1, a lysosomal cysteine protease involved in intracellular protein catabolism. The loss of *Ctsl* leads to a *NPC1*-like phenotype with cholesterol sequestration, suggesting its overexpression could be a compensatory mechanism to the loss of *Npc1* [15].
Ctss			↑									↑		*Ctss* and *Ctsz* encode Cathepsin S and Z which are lysosomal cysteine proteases involved in intracellular protein catabolism and CTSS is known to be increased at the protein level in $Npc1^{-/-}$ mouse tissues [16].
Ctsz			↑									↑		
Fabp5			↑	↑										*Fabp5* encodes Fatty Acid Binding Protein 5 which is involved in fatty acid uptake, transport, and metabolism. May be a compensation mechanism due to the defective lysosomal lipid storage.
Fcer1g			↑											*Fcer1g* encodes for the high affinity immunoglobulin epsilon receptor subunit gamma, a receptor involved in axon regeneration previously reported [17] to be expressed glial cells [18].
Fstl4												↓		*Fstl4* encodes a protein of unknown function: Follistatin Like 4
Fxyd7												↓		*Fxyd7* is encodes FXYD7, a brain specific member of the FXYD family. FXYD7 functions to regulate neuronal excitability by controlling Na,K-ATPase α1–β isozymes K+ affinity [19]. However, the significant change in *Fxyd7* expression is not associated with electrophysiological differences in Purkinje neurons following the loss of *Npc1*[20].
Hexb			↑											*Hexb* encodes hexosaminidase subunit beta which catalyzes the lysosomal degradation of the ganglioside GM2. GM2 is known to accumulate in NPC [21]. Carrying pathogenic variants on both alleles leads to Sandhoff disease. Its expression at the

Table S1 (continued)

Gene	A	ECC	ESC	O	OP	EC	VLC	VSM	B/S	GC	IN	PN	UBC	Gene description and relevance to NPC1
														protein level was previously reported to be increased in *Npc1*$^{-/-}$ tissues [16].
Gfap	↑													*Gfap* encodes one of the major intermediate filament proteins of mature astrocytes known to be overexpressed in reactive astrocytes [22].
Gstp1				↑										*Gstp1* encodes Glutathione S-transferase P1, an enzyme involved in detoxification of reactive species, potentially detrimental, by conjugation with glutathione. Increased expression of GSTP1 has been proposed to be neuroprotective [23].
Lyz2	↑	↑	↑	↑	↑		↑	↑	↑		↑	↑	↑	*Lyz2* gene encodes lysozyme M, an antibacterial protein homologous to human lysozyme. Neuronal expression of lysozyme may be detrimental in Sanfilippo syndrome type B [24].
Manf			↓											*Manf* encodes Mesencephalic Astrocyte Derived Neurotrophic Factor, a neuroprotective factor involved in the control of endoplasmic reticulum stress signaling [25]. Its downregulation is suggested to affect sleep and promote neurodegeneration by promoting neuroinflammation, a hallmark of NPC [4, 26].
Map3k7cl								↑						*Map3k7cl* encodes MAP3 Kinase 7 C-Terminal Like protein. The function of this protein is not known.
Mt1	↑													*Mt1* and *Mt2* are members of the metallothionein family of genes, coding for antioxidant proteins which bind divalent heavy metal ions. These receptors when binding to melatonin have pro-survival and anti-inflammatory functions [27].
Mt2	↑													
Npc2				↑										NPC2 functions in concert with NPC1 to transport unesterified cholesterol out of the endolysosomal compartment [28] and increased expression of *Npc2* has been reported in *Npc1*$^{-/-}$ oligodendrocytes [10].

Table S1 (continued)

Gene	A	ECC	ESC	O	OP	EC	VLC	VSM	B/S	GC	IN	PN	UBC	Gene description and relevance to NPC1
Ptgds				↑										*Ptgds* encodes prostaglandin-H2 D-isomerase (PTGDS), an enzyme that converts prostaglandin H2 to prostaglandin D2. Prostaglandin D2 is a known neuromodulator and trophic factor in the CNS [29] suggesting its overexpression is neuroprotective in an attempt to slow the progressive cerebellar degeneration.
S100a1				↑										*S100a1* encodes S100 calcium-binding protein A1 (S100A1). The function of S100A1 has been extensively studied in the skeletal muscle cells for its calcium dependent interaction with ryanodine 1, a well-established Purkinje neuron marker [30]. Disrupted calcium signaling appears to be involved in NPC cellular pathology and Purkinje cell loss [31-33].
Spp1			↑											*Spp1* encodes the Osteopondin, a protein with anti-apoptotic and immunomodulatory functions [4].
Them6												↑		*Them6* encodes thioesterase superfamily member 6, a protein of unknown function.
Tmsb4x			↑											*Tmsb4x* encodes Thymosin beta-4 a protein of unknown function expressed in the CNS.
Trem2			↑											TREM2 (triggering receptor expressed on myeloid cells 2) is thought to function as a receptor for TYROBP [34]. Mutations of either *TYROBP* or *TREM2* are found in Nasu-Hakola disease, an autosomal recessive disorder characterized by progressive dementia and bone defects [35]. Variants of *TREM2* are risk factors for Alzheimer disease [34]. Increased expression of *Trem2* is associated with neuroinflammation in general [36], but has specifically been observed in microglia from *Npc1*$^{-/-}$ mutant mice [37].

Table S1 (continued)

Gene	A	ECC	ESC	O	OP	EC	VLC	VSM	B/S	GC	IN	PN	UBC	Gene description and relevance to NPC1
Ttr	↑								↑					*Ttr* encodes for transthyretin, a protein present in serum and cerebrospinal fluid which transports thyroid hormone and retinol. TTR misfolding and aggregation is associated with amyloid diseases [38].
Tyrobp		↑	↑		↑		↑					↑	↑	*Tyrobp* encodes TYRO protein tyrosine kinase-binding protein, a transmembrane signaling protein also known as DAP12. This protein appears to have multiple functions including involvement in myelination and [39]. Loss of TYROBP function delays disease progression in Alzheimer Disease mice [40]; however, given its involvement in dementia, it is not clear whether inhibition of DAP12 function would be of potential benefit in NPC1.

3-week-old NPC1 mutant expression

Gene	A	ECC	ESC	O	OP	EC	VLC	VSM	B/S	GC	IN	PN	UBC	Gene description and relevance to NPC1
Slc16a1						↓								*Slc16a1* or solute carrier family 16-member 1 gene encodes for monocarboxylate transporter 1 (MCT1) that mediates the movement of monocarboxylates through the plasma membrane [41]. This overexpression might prevent the shift toward a glycolytic metabolism reported in other cell type following the loss of *Npc1* [37].
Timp3						↑								*Timp3* encodes tissue inhibitor of metalloproteinase 3. Metalloproteinases are a group of enzymes involved in degradation of the extracellular matrix that functions to facilitate cell movement and inflammation.

Table S1 References

1. Wosik, K., et al., *Angiotensin II controls occludin function and is required for blood-brain barrier maintenance: Relevance to multiple sclerosis.* Journal of Neuroscience, 2007. **27**(34): p. 9032-9042.
2. Fu, R., et al., *Apolipoprotein E genotype and neurological disease onset in Niemann-Pick disease, type C1.* Am J Med Genet A, 2012. **158A**(11): p. 2775-80.
3. Liu, C.C., et al., *Apolipoprotein E and Alzheimer disease: risk, mechanisms and therapy.* Nat Rev Neurol, 2013. **9**(2): p. 106-18.
4. Ikeshima-Kataoka, H., *Neuroimmunological Implications of AQP4 in Astrocytes.* Int J Mol Sci, 2016. **17**(8).
5. Caron, C., et al., *CdGAP/ARHGAP31, a Cdc42/Rac1 GTPase regulator, is critical for vascular development and VEGF-mediated angiogenesis.* Sci Rep, 2016. **6**: p. 27485.
6. Isrie, M., et al., *Isolated terminal limb reduction defects: extending the clinical spectrum of Adams-Oliver syndrome and ARHGAP31 mutations.* Am J Med Genet A, 2014. **164A**(6): p. 1576-9.
7. Smith, L.K., et al., *beta2-microglobulin is a systemic pro-aging factor that impairs cognitive function and neurogenesis.* Nat Med, 2015. **21**(8): p. 932-7.
8. Lopez, M.E., A.D. Klein, and M.P. Scott, *Complement is dispensable for neurodegeneration in Niemann-Pick disease type C.* J Neuroinflammation, 2012. **9**: p. 216.
9. Tancini, B., et al., *Insight into the Role of Extracellular Vesicles in Lysosomal Storage Disorders.* Genes (Basel), 2019. **10**(7).
10. Strauss, K., et al., *Exosome secretion ameliorates lysosomal storage of cholesterol in Niemann-Pick type C disease.* J Biol Chem, 2010. **285**(34): p. 26279-88.
11. Sugita, S., et al., *Induction of T regulatory cells by cytotoxic T-lymphocyte antigen-2alpha on corneal endothelial cells.* Invest Ophthalmol Vis Sci, 2011. **52**(5): p. 2598-605.
12. Chung, C., et al., *Genetic and pharmacological evidence implicates cathepsins in Niemann-Pick C cerebellar degeneration.* Human Molecular Genetics, 2016. **25**(7): p. 1434-1446.
13. Koch, S., et al., *Morphologic and functional correlates of synaptic pathology in the cathepsin D knockout mouse model of congenital neuronal ceroid lipofuscinosis.* J Neuropathol Exp Neurol, 2011. **70**(12): p. 1089-96.

Table S1 (continued)

14. Amritraj, A., et al., *Increased activity and altered subcellular distribution of lysosomal enzymes determine neuronal vulnerability in Niemann-Pick type C1-deficient mice.* Am J Pathol, 2009. **175**(6): p. 2540-56.
15. Cermak, S., et al., *Loss of Cathepsin B and L Leads to Lysosomal Dysfunction, NPC-Like Cholesterol Sequestration and Accumulation of the Key Alzheimer's Proteins.* Plos One, 2016. **11**(11).
16. Sleat, D.E., et al., *Proteomic analysis of mouse models of Niemann-Pick C disease reveals alterations in the steady-state levels of lysosomal proteins within the brain.* Proteomics, 2012. **12**(23-24): p. 3499-3509.
17. Martin, K.B., et al., *Identification of Novel Pathways Associated with Patterned Cerebellar Purkinje Neuron Degeneration in Niemann-Pick Disease, Type C1.* Int J Mol Sci, 2019. **21**(1).
18. Zhang, G., et al., *Fcgamma receptor-mediated inflammation inhibits axon regeneration.* PLoS One, 2014. **9**(2): p. e88703.
19. Beguin, P., et al., *FXYD7 is a brain-specific regulator of Na,K-ATPase alpha 1-beta isozymes.* Embo Journal, 2002. **21**(13): p. 3264-3273.
20. Elrick, M.J., et al., *Conditional Niemann-Pick C mice demonstrate cell autonomous Purkinje cell neurodegeneration.* Human Molecular Genetics, 2010. **19**(5): p. 837-847.
21. Vanier, M.T., *Lipid changes in Niemann-Pick disease type C brain: personal experience and review of the literature.* Neurochem Res, 1999. **24**(4): p. 481-9.
22. Liddelow, S.A., et al., *Neurotoxic reactive astrocytes are induced by activated microglia.* Nature, 2017. **541**(7638): p. 481-487.
23. Sun, K.H., et al., *Glutathione-S-transferase P1 is a critical regulator of Cdk5 kinase activity.* J Neurochem, 2011. **118**(5): p. 902-14.
24. Ohmi, K., et al., *Sanfilippo syndrome type B, a lysosomal storage disease, is also a tauopathy.* Proceedings of the National Academy of Sciences of the United States of America, 2009. **106**(20): p. 8332-8337.
25. Renko, J.M., et al., *Mesencephalic Astrocyte-Derived Neurotrophic Factor (MANF) Elevates Stimulus-Evoked Release of Dopamine in Freely-Moving Rats.* Molecular Neurobiology, 2018. **55**(8): p. 6755-6768.
26. Walkowicz, L., et al., *Downregulation of DmMANF in Glial Cells Results in Neurodegeneration and Affects Sleep and Lifespan in Drosophila melanogaster.* Frontiers in Neuroscience, 2017. **11**.
27. Tarocco, A., et al., *Melatonin as a master regulator of cell death and inflammation: molecular mechanisms and clinical implications for newborn care.* Cell Death Dis, 2019. **10**(4): p. 317.
28. Vanier, M.T., *Niemann-Pick disease type C.* Orphanet J Rare Dis, 2010. **5**: p. 16.

Table S1 (continued)

29. Cruz Duarte, P., B. St-Jacques, and W. Ma, *Prostaglandin E2 contributes to the synthesis of brain-derived neurotrophic factor in primary sensory neuron in ganglion explant cultures and in a neuropathic pain model.* Exp Neurol, 2012. **234**(2): p. 466-81.
30. Wright, N.T., et al., *S100A1 and calmodulin compete for the same binding site on ryanodine receptor.* J Biol Chem, 2008. **283**(39): p. 26676-83.
31. Lloyd-Evans, E., et al., *Niemann-Pick disease type C1 is a sphingosine storage disease that causes deregulation of lysosomal calcium.* Nat Med, 2008. **14**(11): p. 1247-55.
32. Yamamoto, T., T. Tokoro, and Y. Eto, *The attenuated elevation of cytoplasmic calcium concentration following the uptake of low density lipoprotein in type C Niemann-Pick fibroblasts.* Biochem Biophys Res Commun, 1994. **198**(2): p. 438-44.
33. Ferrante, A., et al., *The adenosine A2A receptor agonist T1-11 ameliorates neurovisceral symptoms and extends the lifespan of a mouse model of Niemann-Pick type C disease.* Neurobiol Dis, 2018. **110**: p. 1-11.
34. Carmona, S., et al., *The role of TREM2 in Alzheimer's disease and other neurodegenerative disorders.* Lancet Neurol, 2018. **17**(8): p. 721-730.
35. Kaneko, M., et al., *Nasu-Hakola disease: The first case reported by Nasu and review: The 50th Anniversary of Japanese Society of Neuropathology.* Neuropathology, 2010. **30**(5): p. 463-70.
36. Keren-Shaul, H., et al., *A Unique Microglia Type Associated with Restricting Development of Alzheimer's Disease.* Cell, 2017. **169**(7): p. 1276-1290 e17.
37. Cougnoux, A., et al., *Microglia activation in Niemann-Pick disease, type C1 is amendable to therapeutic intervention.* Hum Mol Genet, 2018. **27**(12): p. 2076-2089.
38. Ueda, M. and Y. Ando, *Recent advances in transthyretin amyloidosis therapy.* Transl Neurodegener, 2014. **3**: p. 19.
39. Colonna, M., *DAP12 signaling: from immune cells to bone modeling and brain myelination.* J Clin Invest, 2003. **111**(3): p. 313-4.
40. Audrain, M., et al., *Integrative approach to sporadic Alzheimer's disease: deficiency of TYROBP in a tauopathy mouse model reduces C1q and normalizes clinical phenotype while increasing spread and state of phosphorylation of tau.* Mol Psychiatry, 2019. **24**(9): p. 1383-1397.
41. Al-Khawaga, S., et al., *A SLC16A1 Mutation in an Infant With Ketoacidosis and Neuroimaging Assessment: Expanding the Clinical Spectrum of MCT1 Deficiency.* Front Pediatr, 2019. **7**: p. 299.

Table S3

Primary Antibody	Dilution	Incubation	Application	Host	Company	Clone/Catalog No.
GFAP	1:400	Overnight, 4◦C	IF	Chicken	Novus	NPP1-05198
Calbindin	1:400	Overnight, 4◦C	IF	Mouse	Sigma-Aldrich Millipore	CB-955
Calbindin	1:400	Overnight, 4◦C	IF	Rabbit	Cell Signaling Tech.	13176S
CD117/KIT	1:400	Overnight, 4◦C	IF	Rat	R&D Systems	MAB1356
ALDH1L1	1:100	Overnight, 4◦C	IF	Rabbit	Invitrogen	PA532127
AQP4	1:400	Overnight, 4◦C	IF	Mouse	LS Bio	LS-C413484
IBA1	1:200	Overnight, 4◦C	IF	Rabbit	Wako	019-19741
OLIG2	1:200	Overnight, 4◦C	IF	Rabbit	Sigma-Aldrich Millipore	AB9610
CD68	1:800	Overnight, 4◦C	IF	Rat	Sigma-Aldrich Millipore	FA-11
TBR2	1:400	Overnight, 4◦C	IF	Rabbit	Sigma-Aldrich Millipore	AB2283
AQP1	1:400	Overnight, 4◦C	IF	Mouse	abcam	ab9566
Parvalbumin	1:1000	Overnight, 4◦C	IF	Rabbit	Abcam	ab11427
NeuroD1	1:1000	Overnight, 4◦C	IF	Rabbit	Abcam	ab213725
CBLN3	1:400	30 min, 4◦C	FC	Rabbit	Novusbio	NBP1-85844
TTR	1:400	Overnight, 4◦C	IF	Rabbit		
NEUN	1:400	Overnight, 4◦C	IF	Mouse	Sigma-Aldrich Millipore	A-60
APOE	1:200	30 min, 4◦C	FC	Rabbit	Biodesign International	K23100R
CD11c-FITC	1:100	30 min, 4◦C	FC	Rat	Biolegend	N418
**Secondary Antibody**	**Dilution**	**Incubation**	**Application**	**Host**	**Company**	**Clone/Catalog No.**
anti-Chicken conjugated to Alexa-Fluor 488	1:2000	1 hour, room temp	IF	Goat	ThermoFischer Scientific	A-11039
anti-Mouse conjugated to Alexa-Fluor 488	1:2000	1 hour, room temp	IF	Goat	ThermoFischer Scientific	A28175
anti-Mouse conjugated to DyLight-594	1:2000	1 hour, room temp	IF	Goat	ThermoFischer Scientific	35510
anti-Mouse conjugated to Alexa-Fluor-633	1:2000	1 hour, room temp	IF	Goat	ThermoFischer Scientific	A-21052
anti-Rabbit conjugated to Alexa-Fluor 488	1:2000	1 hour, room temp	IF	Goat	ThermoFischer Scientific	A27034
anti-Rabbit conjugated to AlexaFluor-594	1:2000	1 hour, room temp	IF	Goat	ThermoFischer Scientific	R37119
anti-Rabbit conjugated to Alexa-Fluor-633	1:2000	1 hour, room temp	IF	Goat	ThermoFischer Scientific	A-21071
anti-Rabbit conjugated to Alexa-Fluor 488	1:2000	1 hour, 4◦C	FC	Goat	ThermoFischer Scientific	A27034

Table S3 (continued)

**Reagent**	**Dilution**	**Incubation**	**Application**	**Host**	**Company**	**Clone/Catalog No.**
Hoechst 3342	1:5000	15 min, room temp	IF	N/A	ThermoFischer Scientific	62249
Goat Serum	1:20	1 hour, 4^0C	IF		Sigma-Aldrich Millipore	G9023
Neurotrace	1:1000	1 hour, 4^0C	IF		ThermoFischer Scientific	N21483
30% Bovine Serum Albumin	1:10	10 min, 4^0C	FACS	Bovine	Sigma-Aldrich Millipore	A9576-50ML
TruStain FcX	1:100	10 min, 4^0C	FACS	Rat	Biolegend	101320
Mouse B2M / Beta 2 Microglobulin ELISA Kit			ELISA		LSBio	LS-F14141
Human Lysozyme ELISA Kit			ELISA		LSBio	NBP2-60511
Human CTSD / Cathepsin D ELISA Kit			ELISA		LSBio	LS-F6862-1
Bovine Serum Albumin	2% w/v		WB		Sigma-Aldrich Millipore	A7030
Paraformaldehyde	4% w/v		IF		Sigma-Aldrich Millipore	A6148
Papain dissociation system			Cerebellum dissociation		Worington	LK003150
KAPA Taq Ready Mix			Genotyping		Sigma-Aldrich Millipore	KK1006
PLX3397	290mg/kg	Mouse feed. RT	Microglia depletion		Selleckchem	S7818
**Software**					**Company**	**Version**
Prism 5					Graphpad	Version 5.01
R/Rstudio					CRAN foundation	Version 1.1.463
MS Office 365 and 2016					Microsoft	
ZEN2(Blue)					Zeiss	
ImageJ					NIH	Version 1.52i

**Primers**	**Sequence**	**Company**
Npc1_F	GGACAGCCAAGTAGGCGAC	Integrated DNA Technologies
Npc1_R	GACGTGTGCACCCCCTTTC	Integrated DNA Technologies
